# Influence of Crude Oil Production Chemicals on the Performance of Commercial Demulsifiers

**DOI:** 10.1021/acsomega.5c13209

**Published:** 2026-03-16

**Authors:** José V. L. da Silva, Paulo Cristiano S. Rocha, Marcela R. Ferreira, Rita C. P. Nunes, Elizabete F. Lucas

**Affiliations:** † 28125 Federal University of Rio de Janeiro, Macromolecules Institute, Rua Moniz Aragão 360, Block 8G/CT2, Rio de Janeiro, RJ 21941594, Brazil; ‡ Federal University of Rio de Janeiro, Metallurgical and Materials Engineering Program, COPPE/LADPOL Av. Horácio Macedo, 2030, Block F, Rio de Janeiro, RJ 21941598, Brazil

## Abstract

During oil production, crude oil is commonly recovered as water-in-oil (w/o) emulsions, which require the chemical destabilization of the emulsion for phase separation. Despite the extensive use of production chemicals, their combined effects on emulsion stability and produced water quality in extra-heavy oils are still poorly understood. In this work, the influence of a corrosion inhibitor (*I*_cor_), a scale inhibitor (*I*_sca_), and two enhanced oil recovery (EOR) polymers (SPAM and HPAM) on the performance of three commercial emulsion destabilizers (*D*_1_, *D*_2_, and *D*_3_) was systematically investigated using synthetic emulsions prepared with asphaltic crude oil and synthetic brine (∼90,000 ppm). Bottle tests showed that, in the absence of EOR polymers, D_1_, D_2_, and D_3_ achieved water separation of ∼55%, 58%, and 78%, respectively, at high concentrations. The individual or combined addition of I_cor_ and I_sca_ did not induce phase separation (0.0% water separated) and did not significantly affect the demulsifiers’ performance. In contrast, the presence of EOR polymers reduced demulsification efficiency, with HPAM showing the most pronounced effect, decreasing the maximum water separation to ∼39%, 45%, and 59% for *D*_1_, *D*_2_, and *D*_3_, respectively. The method of additive addition did not significantly alter the separation efficiency. Interfacial tension measurements decreased from ∼27.6 mN/m (without additives) to ∼13.2–11.6 mN/m in the presence of demulsifiers, while smaller reductions were observed when EOR polymers were present, indicating competitive adsorption at the oil–water interface. Droplet size analysis revealed a direct correlation between increased water separation and larger droplet values, with D90 values reaching ∼1900 μm at high demulsifier concentrations. Although inhibitors did not affect separation efficiency, enhanced oil recovery (EOR) polymers significantly reduced the quality of the produced water, increasing the total oil and grease (TOG) content from ∼36 to ∼410 ppm (HPAM) and ∼600 ppm (SPAM). These results demonstrate that EOR polymers impair both the efficiency of emulsion destabilization and the quality of produced water in extra-heavy oil systems, while inhibitors showed no effect on the emulsion destabilization performance.

## Graphical abstract

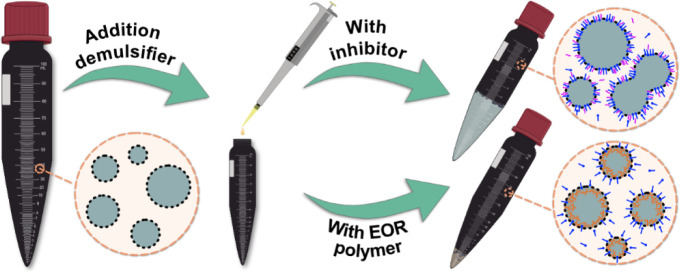

## 1 Introduction

The conditions of the oil production process, such as shear, induce mixing of the oil and aqueous phases, leading to the formation of emulsions.^1–3^ The presence of some natural constituents of oil, such as asphaltenes, resins, and salts dissolved in brine, as well as solid particles derived from the reservoir rock, contributes substantially to this stabilization through phenomena such as the Gibbs–Marangoni effect, steric repulsion, and electrostatic repulsion.^4–10^ In extra-heavy crude oils, this effect is accentuated due to the high concentration of asphaltenes, which present resistant layers at the oil–water interface, where they adsorb forming rigid, cohesive, and viscoelastic interfacial films. These films significantly increase the stability of water-in-oil emulsions, inhibiting the coalescence of water droplets and promoting the retention of corrosive species such as CO_2_, H_2_S, organic acids, and salts, thereby intensifying corrosion processes under deposits and the general corrosion mechanisms in production systems.^11,12^ Moreover, the injection of chemicals used in the enhanced recovery process alters the properties of the w/o interface, making these emulsions even more stable through a synergistic effect with the oil’s stabilizing components, the mechanism of which is unknown.^13–18^

Approximately 80% of crude oil is recovered in the form of w/o emulsions.^19^ This phenomenon can cause logistical and economic problems, mainly related to flow assurance, such as increased oil viscosity and corrosion of production lines.^20,21^ To address these issues, crude oil is subjected to a demulsification process as soon as it reaches the surface, through techniques such as heating, application of electric fields, centrifugation, membrane separation, and, most notably, the use of chemical additives.^22,23^ Among the available substances, nonionic surfactants such as polyalkylene glycol, nonylphenol ethoxylate, polyamine, polyamide, and resin alkoxylates have been widely used to promote demulsification through interfacial destabilization mechanisms. These surfactants are adsorbed at the water–oil interface, displacing the natural surfactants and facilitating droplet coalescence and phase separation.^3,24–30^

Recent research suggests that some additives used in production, such as enhanced oil recovery (EOR) additives and scale inhibitors, also increase the stability of w/o emulsions by affecting the separation effectiveness of demulsifiers.^31–35^

Lin et al. (2008) investigated the influence of EOR polymers on the stability and demulsification of w/o emulsions. In this context, three polymers were evaluated—HPAM 3530S, AX-74H, and a hydrophobic association polymer (HAP)—at 60 ppm in synthetic emulsions (60/40 v:v) through bottle tests. Among the results, AX-74H and HAP slowed the water-separation kinetics, an effect attributed to the increase in viscosity and surfactant-induced droplet stabilization, although the final water separation after 1 h of testing was comparable to that of the system without additives. In contrast, HPAM 3530S led to greater water separation, indicating that the demulsification process was not compromised by the addition of chemical products.^18^ Another study evaluated the effect of two polymers (FLOPAAM 3230S and 3630S) on high-salinity synthetic brine/heavy crude oil emulsions by determining the droplet-size distribution, electrocoalescence, and interfacial electrorheology. These tests indicated that HPAMs alone did not significantly influence emulsion destabilization, although their presence reduced free water release when combined with demulsifiers. Increased resistance to electrocoalescence and higher interfacial viscosity were observed, suggesting that the EOR polymers may interfere with demulsifier efficiency through competitive adsorption at the oil–water interface.^36^

Other chemicals widely used in oil production are wax deposition inhibitors, with ethylene-vinyl acetate (EVA) copolymers possibly being the most studied.^37–40^ The effect of 4 samples of EVA (EVA 10, EVA 20, EVA 30, and EVA 40, with vinyl acetate contents of 10, 20, 30, and 40% wt%, respectively) on the stability of model emulsions containing asphaltenes and resins was analyzed. By using bottle tests under the conditions of the demulsification process, the authors observed that the increase in polarity of EVA contributed to the reduction of emulsion stability by weakening the interfacial film, due to the more polar EVA’s ability to disperse the asphaltenes at the interface.^41^ Since the most efficient EVA samples, such as paraffin deposition inhibitors, are those with a vinyl acetate content of around 30% wt%, the presence of this additive would not pose a problem for the demulsification step.^42^

In addition to the increased emulsion stability caused by the additives used in production, there are also concerns regarding the quality of the separated water, which often results in the formation of another emulsion along with dispersed free oil.^43^ Ferraz et al. (2023) evaluated the influence of partially hydrolyzed polyacrylamide (HPAM) on the stability of 60/40 w/o v:v synthetic emulsions and on the quality of the separated water after testing in bottles, with and without a commercial demulsifier. The results revealed that the presence of the EOR additive caused a reduction in the volume of separated water and higher oil and grease content (TOG) values for the separated water. According to the authors, this occurred because HPAM has an amphiphilic structure with lower HLB. Thus, in w/o emulsions, the molecules can reduce the action of demulsifiers at the interface, and in o/w emulsions, the low-HLB structure tends to stabilize the oil-in-water system after oil treatment.^44^

Recently, the dual functionality of polymers as demulsifiers and corrosion inhibitors has been investigated. Block copolymers synthesized from oleic imidazoline, ethylene oxide, and propylene oxide—called OIPE—were evaluated in chemical demulsification tests and compared with the commercial polyether-based demulsifier SP169. The copolymer with a 60:40 oleic imidazoline/propylene oxide:ethylene oxide ratio had superior performance, achieving 90% water separation in 1 h, compared to 80% for the commercial additive. Despite its greater separation efficiency, the separated aqueous phase had a darker appearance, indicating a reduction in the quality of the separated water. According to the authors, this may have been related to the adsorption of imidazoline groups at the oil–water interface, potentially contributing to the formation of residual o/w emulsions derived from the separated water together with the dispersed free oil.^45^

Recent findings highlight that production chemicals with interfacial activity can simultaneously affect the efficiency of emulsion destabilization and impair the quality of separated water due to complex adsorption phenomena at the oil–water interface.^16–18,41,44^ However, the studies available in the literature have almost exclusively investigated conventional crude oils, neglecting extra-heavy systems, in which interfacial interactions are markedly intensified by high viscosity and high asphaltene content. Moreover, no research we found evaluated the influence of chemical products used together, which reproduces the crude oil production scenario. This gap is particularly critical from an industrial standpoint, since heavy and extra-heavy crude oils represent a significant fraction of the world’s remaining petroleum resources and are expected to dominate future production scenarios. Despite this, the combined influence of commonly applied production chemicals on the performance of commercial emulsion destabilizers and the quality of separated water in extra-heavy crude oil emulsions remains largely unexplored. For this reason, the main objective of this study was to investigate the influence of production chemicals (enhanced recovery additives, scale, and corrosion inhibitors) added individually and in combination on the performance of commercial demulsifiers using synthetic emulsions of brine in asphaltic crude oil. We also investigated the correlations between emulsion stability, interfacial tension, and droplet size. Finally, the TOG content of the separated water after the demulsification test was determined to verify the influence of chemicals on water quality.

## 2 Materials and Methods

### 2.1 Materials

A sample of Brazilian asphaltic crude oil with an API gravity of 13.2, a water content of 2.8 wt %, and an asphaltene content of 12.9 wt % was provided by the company Equinor (Brazil). Sodium bicarbonate (99.7%), potassium chloride (99%), sodium chloride (99.5%), acetic acid (99.7%), and mineral oil were supplied by Isofar Indústria e Comércio de Produtos Químicos Ltda (Duque de Caxias, Brazil). Barium chloride dihydrate (99%), calcium chloride dihydrate (99%), magnesium chloride hexahydrate (99%), hydrochloric acid (37%), and sodium hydroxide (99%) were purchased from Labsynth Produtos para Laboratórios Ltda. (Diadema, Brazil). Strontium chloride hexahydrate (99%) was supplied by Vetec Química Fina (Duque de Caxias, Brazil), and silicon dioxide (99%) was provided by Merck S.A. (Rio de Janeiro, Brazil).

Partially hydrolyzed polyacrylamide (FLOPAAM 3630) with a molar mass of 20,000 000 Da, named HPAM in this study; sulfonated polyacrylamide (FLOPAAM AN125) with a molar mass of 8,000 000 Da, called SPAM; three different demulsifiers, designated *D*_1_, *D*_2_, and *D*_3;_ a scale inhibitor (*I*_sca_); and a corrosion inhibitor (*I*_cor_) were supplied by Equinor (Brazil). All of these are commercial additives. The compositions of these chemicals are subject to confidentiality restrictions.

### 2.2 Experimental Procedure

#### 2.2.1 Preparation of Synthetic Brine

The brine was prepared with a total salt concentration of approximately 90,000 ppm. The salts were dissolved in deionized water under magnetic stirring at room temperature, in the following order of addition: 80,800 mg/L of NaCl; 8,730 mg/L of CaCl_2_·2H_2_O; 200 mg/L of NaHCO_3_; 4,340 mg/L of MgCl_6_·6H_2_O; 810 mg/L of KCl; 720 mg/L of SrCl_2_·6H_2_O; 35 mg/L of BaCl_6_·2H_2_O; and 25 mg/L of SiO_2_. After dissolution, glacial acetic acid was added at a concentration of 290 μL/L, and the pH was adjusted to 7 using HCl and NaOH.

For tests involving the EOR polymers, SPAM or HPAM were added at concentrations of 300, 450, or 900 mg/L. The solution was kept under magnetic stirring and heated at 80 °C for 90 min to solubilize the polymer.

#### 2.2.2 Preparation of Synthetic Brine-in-Oil Emulsions

Emulsions were prepared using synthetic brine, EOR polymers (SPAM or HPAM), asphaltic crude oil, and demulsifiers following a controlled and reproducible procedure. Initially, the brine was prepared at a concentration of approximately 90,000 ppm. When necessary, SPAM or HPAM was dissolved in brine under magnetic stirring for 90 min at 80 °C.

Before the formation of the w/o emulsion, the aqueous and oily phases were heated separately in an oven at 80 °C for 30 min to reduce the viscosity of the crude oil and facilitate mixing. Subsequently, the brine was slowly added to the crude oil under continuous manual stirring with a glass rod at room temperature for 8 min. The resulting mixture was then subjected to mechanical stirring using a Polytron PT 10–35GT homogenizer (Kinematica) at 15,000 rpm for 6 min. During this step, external heating was applied in an oil bath at 60 °C to maintain the phases at an elevated temperature due to the viscosity of the phases and thus obtain a w/o emulsion.

The samples were then transferred to graduated bottles and placed in a thermostatic bath at 100 °C for 20 min to reach the stability test temperature. When applicable, the demulsifier was then added using a micropipette, followed by manual stirring for 1 min to ensure uniform distribution of the additive throughout the emulsion. Preliminary studies were conducted using w/o volume ratios of 40/60, 50/50, and 60/40 (v/v), taking into account the water content originally present in the crude oil.

Other kinds of emulsions were also prepared for comparison: without demulsifier and without SPAM or HPAM; with demulsifier and without SPAM or HPAM; with SPAM or HPAM and without demulsifier.

#### 2.2.3 Emulsion Stability by Bottle Test

After preparing the emulsions, the conical tubes were placed back in the thermostatic bath at 100 °C and immediately began the test. The separated water volume was recorded every 5 min for the first 30 min and every 10 min for the final 30 min, obtaining the demulsification kinetics. However, the data reported in this work were obtained at the end of the test, after 60 min, when the amount of water separated became practically constant. The temperature and time parameters of the analysis were chosen considering the actual separation conditions in the Brazilian extra-heavy oil production field and also the studies by Ferraz et al. (2022) and Nunes et al. (2022), who used the flask test and extra-heavy oil under specific conditions.^46,47^ Moreover, all analyses were performed in duplicate, aiming at the reproducibility and reliability of the results.

The concentrations of chemicals, added individually and together, were defined based on those used in a Brazilian oil field, as presented in Table 1.

**Table 1:** Concentration of Chemicals Used in Bottle Test

		Chemical concentration (ppm)				
Concentration	Test type	*D*_1_	*D*_2_	*D*_3_	*I*_cor_	*I*_sca_
Low	Individually	50	0	0	0	0
		0	65	0	0	0
		0	0	50	0	0
		0	0	0	80	0
		0	0	0	0	40
High		650	0	0	0	0
		0	845	0	0	0
		0	0	650	0	0
		0	0	0	1040	0
		0	0	0	0	520
Low	Together	50	65	50	80	40
High		650	845	650	1040	520

##### 2.2.3.1 Chemicals Added Individually at Low and High Concentrations

The tests involved the addition of demulsifiers (*D*_1_, *D*_2_, or *D*_3_) and inhibitors (*I*_cor_ or *I*_sca_) at low and high concentrations individually in the presence or absence of SPAM or HPAM.

##### 2.2.3.2 Chemicals Added Together at Low Concentrations

For this test, two procedures were employed:

Procedure (A): simultaneous addition of additives (*D*_1_ + *D*_2_ + *I*_cor_ + *D*_3_ + *I*_sca_). Total test time: 60 min.

Procedure (B): Sequential addition of additives. Demulsifier D_1_ was initially added to the emulsion, followed by manual stirring for 1 min. The tube was then placed in the thermostatic bath at 100 °C for 1 h, after which the volume of separated water was recorded. This procedure was subsequently repeated, first with the addition of *D*_2_ and *I*_cor_ together, and then with the addition of *D*_3_, *I*_cor_, and *I*_sca_ also together. The total duration of the sequential addition tests was 3 h.

#### 2.2.4 Determination of Total Oil and Grease (TOG) by Fluorimetry

The quality of the separated water collected at the end of the bottle test was verified by TOG measurements using a TD-3100 benchtop fluorimeter (Turner Designs), considering the TOG to be inversely proportional to water quality. Because the TOG determination requires a minimum volume of 45 mL, three to four bottles were prepared with the same emulsion composition under identical conditions to obtain the required volume of water.

TOG determination by fluorimetry using water samples collected at the end of the bottle test was performed as follows. A volume of 45 mL of the separated water was removed from the conical tube and transferred to a separation funnel, followed by the addition of 5 mL of *n*-hexane (extraction solvent). The system was agitated manually for 1 min and allowed to rest for 2 min to promote phase separation. The resulting heterogeneous mixture was transferred to a clean conical tube and centrifuged for 5 min at 2,400 rpm to improve phase separation. Subsequently, the sample was returned to the separation funnel, where the aqueous phase was drained and discarded. A 1 mL aliquot of the organic phase was then transferred to a 5 mL volumetric flask, which was filled with *n*-hexane. Finally, the organic phase was analyzed by fluorimetry to quantify the TOG.

The calibration of the device was carried out at two points: one reading of the pure solvent (blank, concentration point of 0.00 ppm) and the other reading of the maximum concentration of the curve (oil in *n*-hexane at 225 ppm). The setup value was 25 ppm, corresponding to 1/9 of the 225 ppm, since the extraction is done with 1 part of *n*-hexane and 9 parts of o/w emulsion. It is important to highlight that this calibration is valid only for the crude oil and solvent used in this study. Therefore, a new calibration is required when changing the type of crude oil or solvent. All analyses were performed in duplicate.

#### 2.2.5 Drop Size and Drop Size Distribution According to Laser Diffraction

To determine the size and size distribution of the droplets, a Mastersizer 3000 (Malvern) with a Hydro EV accessory was used.^48–51^ For the test, the oil phase obtained from the bottle test was initially mixed with mineral oil under magnetic stirring at 80 °C to reduce viscosity and achieve a homogeneous dispersion before inserting it into the equipment. The diluted sample had to reach an opacity of ∼10%. Then, automatic measurements were performed, and the droplet size distribution was obtained as a percentage of volume as a function of the droplet diameter (μm). The values *D*(10), *D*(50), and *D*(90) indicated that 10%, 50%, and 90% of the particles were smaller than those sizes, respectively.

Measurements were performed immediately after: preparing the emulsion, 20 min in a thermostatic bath, and finishing the bottle test. The analyses were carried out in duplicate.

#### 2.2.6 Interfacial Tension by Force Tensiometry

Interfacial tension tests were conducted using a Sigma 700 tensiometer (Attension) with a Wilhelmy plate accessory.^52–54^ When using SPAM or HPAM, the preparation of the aqueous phase, both without and with EOR polymers, followed the procedures described in section 2.2.1. When using demulsifiers and/or inhibitors, the additives were incorporated into the asphaltic petroleum by manual stirring for 1 min and then left to rest for 12 h, according to the concentrations in Table 1. For the interfacial tension measurements, the aqueous and oil phases were preheated in an oven at 80 °C for 30 min to reduce oil viscosity. Then, 25 mL of the aqueous phase was added to the cuvette, placing the Wilhelmy plate in the aqueous phase near the surface, and 25 mL of the oil phase were poured over the aqueous phase. The equipment was programmed to perform the analysis automatically, with interfacial tension readings taken every minute for 30 min. The curves showed an exponential profile, associated with the adsorption process of interfacially active species at the interface. In the beginning, the interfacial tension decreased and then remained constant until the end of the test, evidencing the equilibrium regime. The interfacial tension values were reported as the average of all measurements throughout the whole test. All analyses were conducted in duplicate.

## 3 Results and Discussion

### 3.1 Preliminary Tests to Determine the Optimal W/O Proportion

Since the study focused on oil fields with EOR operations, a high volume of produced water is expected, which increases the likelihood of the formation of w/o emulsions with high water content, especially when heavy crude oil has a high concentration of asphaltenes. Therefore, the aim of the preliminary tests was to determine the emulsion composition with the highest water content while remaining kinetically stable.

To determine the best conditions for emulsion stability tests using the demulsifiers and EOR polymers, preliminary tests were conducted with w/o proportions of 40/60, 50/50, and 60/40 v/v. All emulsions were very stable, with no amount of water separated without a demulsifier. With the addition of 350 ppm of demulsifier (*D*_1_), the percentages of separated water were 7.5%, 45.0%, and 70.0% at the end of the test for the proportions 40/60, 50/50, and 60/40 (v/v), respectively. To study the performance of the demulsifier, the 50/50 emulsion was used, since the 40/60 emulsion was very stable and the 60/40 emulsion was very unstable. Therefore, the 50/50 ratio was selected because it produces a water/oil emulsion with sufficient stability, remaining responsive to the action of the demulsifier, allowing for the evaluation of efficiency based on the volume of water separated. This condition also makes it possible to assess the influence of other production chemicals on the demulsifiers’ performance under representative operating conditions.

To evaluate the influence of the EOR polymers on emulsion stability, tests were conducted using the 50/50 (w/o) proportion. Under these conditions, stable emulsions were obtained without phase separation at 0 and 300 ppm SPAM or HPAM. However, when the concentrations of EOR polymers were increased to 450 and 900 ppm, emulsion formation was not observed. This factor was associated with the increase in the viscosity of the aqueous phase, which made it difficult for the dispersion of the aqueous droplets in the oily phase, which also has high viscosity, preventing the W/O emulsion formation.^55^ Thus, to evaluate the influence of the production chemicals individually and together at low and high concentrations on the performance of the demulsifier, the proportion of 50/50 (w/o) and a concentration of 300 ppm of the EOR polymers were employed. Since it is not possible to use real produced emulsions presenting the same characteristics throughout the whole study, synthetic emulsions were selected. Real produced fluids may present high compositional variability, unknown operational history, and aging impact. Synthetic emulsions have proven to be suitable for systematic evaluation and quantification of stabilization and destabilization phenomena, allowing for the assessment of the influence of production chemicals on w/o emulsions. Indeed, synthetic systems do not completely reproduce field emulsions; however, the behavioral tendencies observed using synthetic emulsions can be extrapolated to real operating conditions.

### 3.2 Influence of Chemicals Added Individually, without and with SPAM or HPAM, on the Emulsion Stability and TOG of the Water Separated

This study was conducted using commercial chemical products at low and high concentrations (Table 1).

#### 3.2.1 Chemicals Added Individually at Low Concentrations

For low concentrations of chemicals without SPAM or HPAM in brine, *D*_3_ (50 ppm) was the most efficient, with 24.0% separated water. *D*_1_ (50 ppm) and *D*_2_ (65 ppm) performed similarly: 8.5% and 7.5% separated water, respectively (Figure 1). As expected, *I*_sca_ and *I*_cor_ did not achieve any phase separation

**Figure 1:**
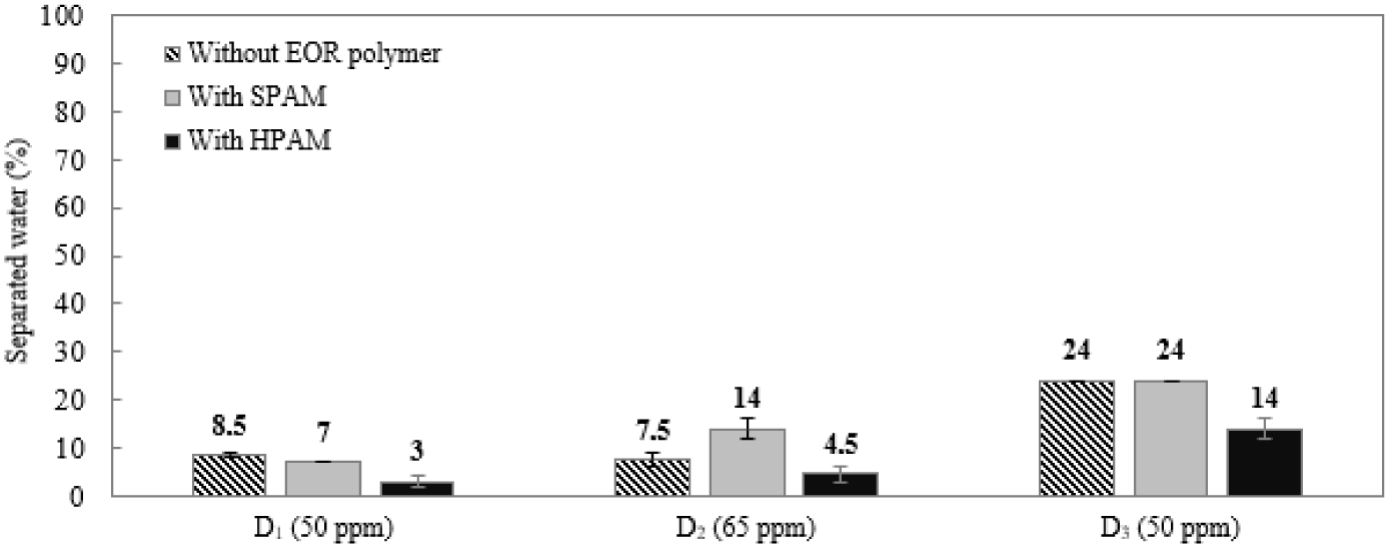
Percentage of separated water for the emulsion without (black hatched bars) and SPAM (gray bars) or HPAM (black bars) in brine, with different demulsifiers (*D*_1_, *D*_2_ and *D*_3_) added individually at low concentrations.

For the emulsions containing SPAM in brine, *D*_1_ and *D*_3_ presented very similar behavior to that observed for the emulsions without the EOR polymer: with 8.5% and 24.0% (without SPAM) and 7.0% and 24.0% (with SPAM), respectively. *D*_2_ in the presence of SPAM was more efficient than in the sample without the EOR polymer: 14.0% and 7.5%, respectively. Again, *I*_sca_ and *I*_cor_ failed to promote phase separation because these inhibitors are surfactant-like molecules with hydrophilic–lipophilic balance (HLB) values that are unsuitable for acting as demulsifiers in w/o systems. The HLB reflects a molecule’s relative affinity for the aqueous and oily phases; compounds with higher HLB values are predominantly hydrophilic and preferentially partition into the aqueous phase. Scale and corrosion inhibitors, such as *I*_sca_ and *I*_cor_, are typically formulated with relatively high HLB values to ensure solubility and activity in the aqueous phase, where they perform their intended function. Consequently, these molecules do not effectively displace stabilizing species, such as asphaltenes and resins, from the interface, making it unlikely that they will induce droplet coalescence and phase separation in w/o emulsions.

For the emulsions prepared with HPAM, the demulsifiers caused a significant reduction in demulsifier performance compared with the systems without EOR polymer and with SPAM. The percentages of separated water were 3.0%, 4.5%, and 14.0% for *D*_1_ (50 ppm), *D*_2_ (65 ppm), and *D*_3_ (50 ppm), respectively. Again, *D*_3_ presented the best performance, confirming the results obtained for the emulsions without and with SPAM. *I*_sca_ and *I*_cor_ as well as the emulsion without the EOR polymer or SPAM, did not cause any phase separation. Figure 2 shows the conical tubes at the end of the bottle test for samples with the chemicals (*D*_1_, *D*_2_, *D*_3_, *I*_sca_, and *I*_cor_) individually added at low concentration, without and with the EOR polymers.

**Figure 2:**
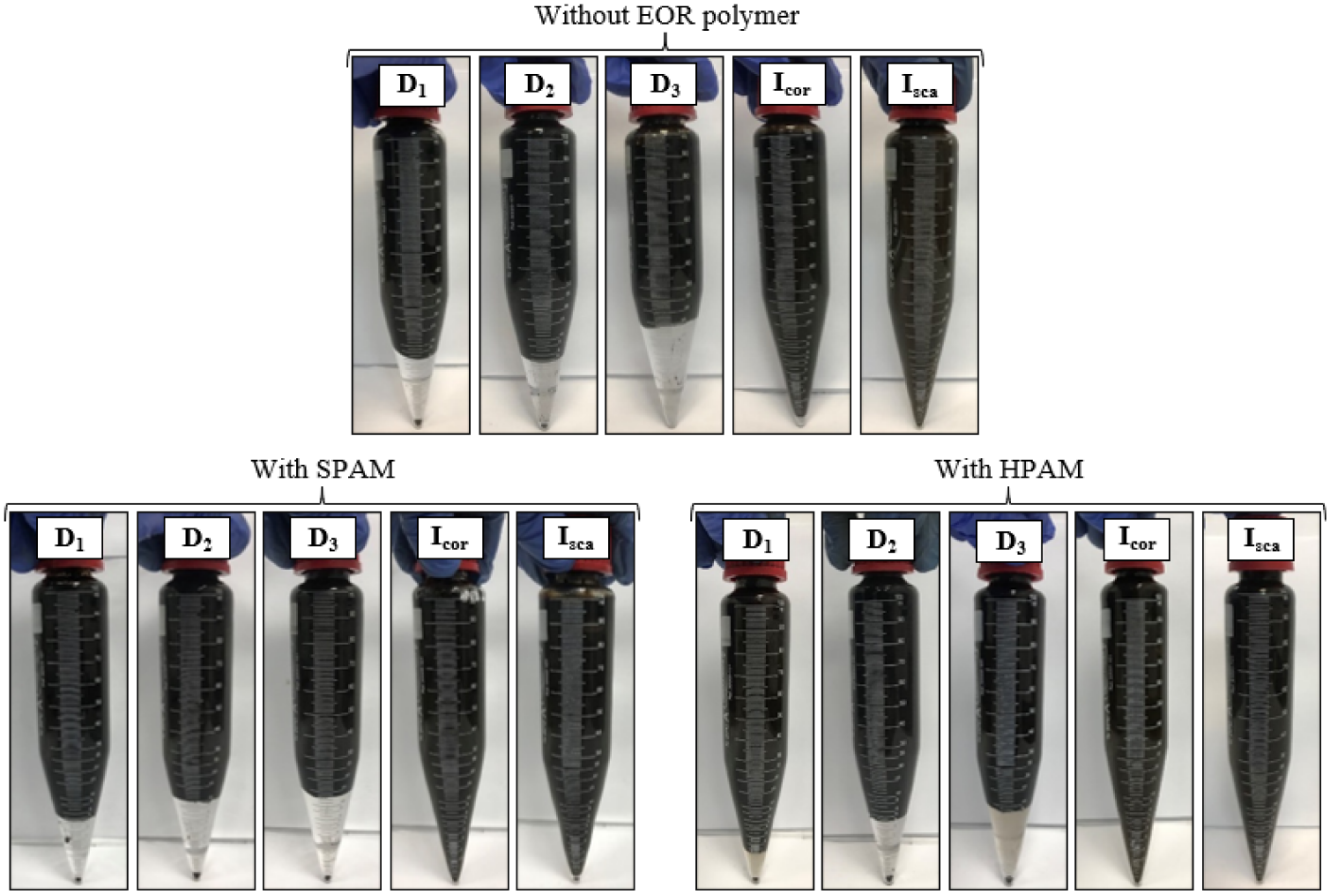
Conical tubes at the end of the test and bottle without EOR polymer, with SPAM or HPAM in brine, with additives *D*_1_, *D*_2_, *D*_3_, *I*_cor_, or *I*_sca_ added individually in low concentrations.

#### 3.2.2 Chemicals Added Individually at High Concentrations

When using a high concentration of demulsifier (Figure 3), an increase in demulsification efficiency was observed, as expected,compared with tests performed at low concentrations (Figure 1). Without the EOR polymer, the volumes of separated water were 55.0%, 58.0%, and 78.0% for demulsifiers *D*_1_, *D*_2_, and *D*_3_, respectively. In the tests with SPAM and HPAM, there was a reduction in demulsification efficiency, with this influence being more significant for HPAM. Considering the composition of petroleum, rich in heavy fraction components such as asphaltenes, the reduction in the efficiency of emulsion destabilization in the presence of enhanced oil recovery (EOR) polymers may be associated with molecules that interact with the active components in emulsion stabilization, as already reported in the literature by Duan et al. (2017). Thus, the formation of the polar EOR/asphaltene polymer complex inhibits the destabilizing action of the emulsion by occupying the interface, the location where demulsifiers act to separate the phases.^56^

**Figure 3:**
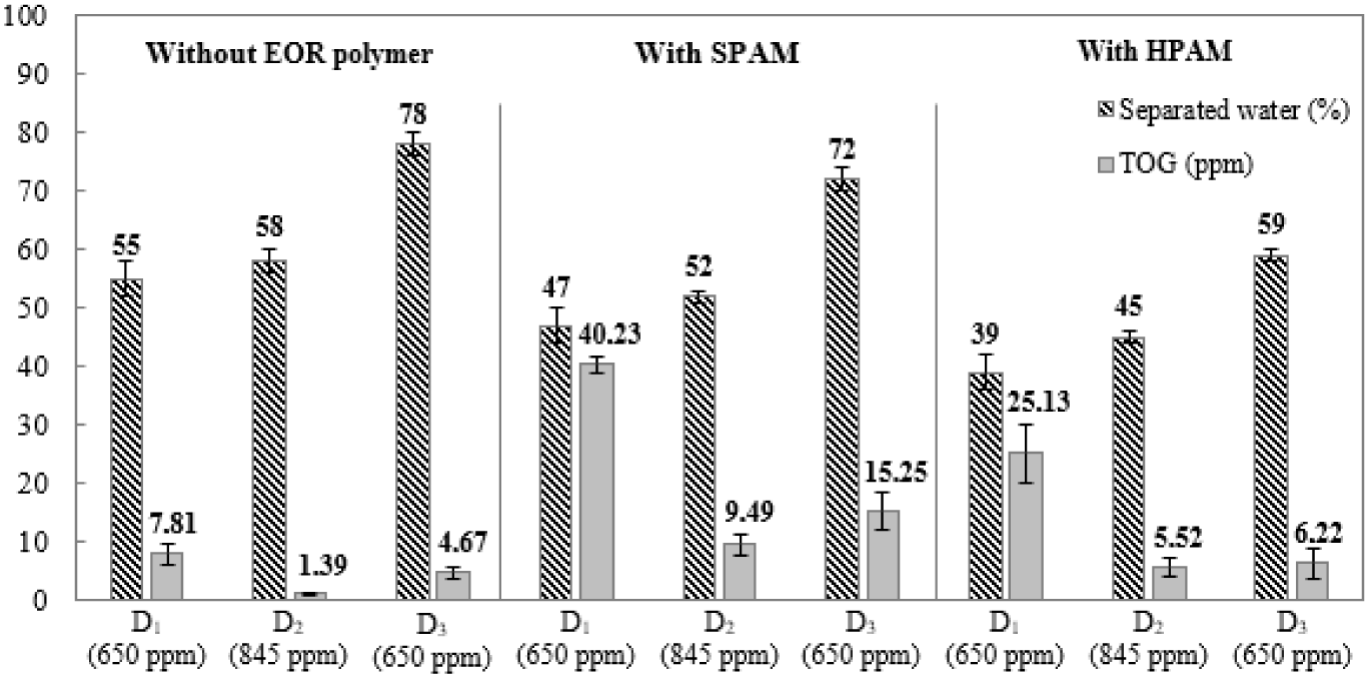
Percentage of separated water for the emulsion (black hatched bars) and TOG of the water separated (gray bars) values of the tests involving the demulsifiers individually at high concentrations, without and with SPAM or HPAM.

Similar to the tests at low concentrations, *D*_3_ presented better performance in comparison with *D*_1_ and *D*_2_ (black hatched bars). Furthermore, under the evaluated conditions, tests with *I*_cor_ and *I*_sca_ at high concentrations, in both the presence and absence of SPAM or HPAM, did not promote any water separation. Considering that TOG analysis by fluorimetry requires a minimum volume of 45 mL of aqueous phase, it was not possible to perform these tests. Thus, the tests in the flask that would originate the samples for analysis showed, at the end of the experiment, a separated water volume equal to 0.0 mL, making the execution of the test unfeasible.

In the TOG tests of the water separated with the different production chemicals (demulsifiers and EOR polymers), their influence can be noted as gray bars. In particular, the water separated from the samples without the EOR polymer presented low TOG values: 7.81, 1.39, and 4.67 ppm when *D*_1_, *D*_2_, and *D*_3_ were used, respectively.

However, the TOG values increased significantly in the water separated from the emulsions containing SPAM: 40.23, 9.49, and 15.25 ppm when *D*_1_, *D*_2_, and *D*_3_ were used, respectively. The polymer could interact with the oil, stabilizing the o/w emulsion and decreasing the water quality.

Similar behavior was also observed for the systems containing HPAM. The TOG values were higher than those observed for the emulsions without the EOR polymer, although the values were lower than those observed for the emulsions containing SPAM. Of particular note, the highest TOG value was observed when *D*_1_ was used in all systems. Therefore, the addition of *D*_1_ to the emulsion containing SPAM produced separated water with the highest TOG (40.23 ppm).

### 3.3 Influence of Chemicals Added Together, without and with SPAM or HPAM, on the Emulsion Stability and TOG of the Water Separated

#### 3.3.1 Chemicals Added Together at Low Concentrations

After evaluating individual additions in the emulsions, the chemicals were added together. The stability was analyzed by a bottle test using two different procedures, as cited in the experimental part.

First, when analyzing Procedure (A), where all the chemicals were added at the same time, the percentages of separated water for low-concentration chemicals were very close for the emulsions prepared without and with SPAM (49.0% and 55.0%, respectively). For the emulsion containing HPAM in brine, the amount of separated water was lower (32.0%). Similar behavior was observed when the chemicals were added individually, confirming the different influences of SPAM and HPAM on the efficiency of the demulsification process.

By comparing the total percentage of separated water when adding demulsifiers individually and all demulsifiers together at the same concentrations (low concentrations) and test time (1 h), synergism occurred among the demulsifiers, as the total percentages of separated water were slightly higher when adding all chemicals together. Furthermore, when compared the separated water values of the tests without and with the corrosion and scale inhibitors together with the different demulsifiers (all chemicals together), there was no significant difference in the results, as can be seen in the data summarized in Table 2. These results reaffirm the demulsification inefficiency of the inhibitor chemicals, as seen in the tests with the same chemicals individually. In particular, the differences were not within the error range of the analyses.

**Table 2:** Comparison between percentages of separated water in emulsion by adding demulsifiers individually, together at low concentration and with all chemicals added together, preparing emulsions without EOR polymer, SPAM, or HPAM

	Percentages of total separated water (**%**)		
	Without EOR polymer	With SPAM	With HPAM
**Sum of demulsifiers added individually**	40.0 ± 2.0	45.0 ± 2.0	21.5 ± 2.5
**Demulsifiers added together**	46.0 ± 2.0	51.0 ± 3.0	30.0 ± 2.0
**All chemicals added together**	49.0 ± 3.0	55.0 ± 1.0	32.0 ± 2.0

In the case of Procedure (B), where the chemicals were added in the same flask following three different steps, greater phase separation was achieved than in Procedure (A), for all types of emulsions: without the EOR polymer and with SPAM or HPAM. However, when Procedure (A) was carried out for 3 h, the percentages of separated water were within the error range for emulsions prepared using HPAM in brine, as shown in Table 3. The emulsion without the EOR polymer produced a small amount of separated water when all additives were added together, even when conducting the test for 3 h. The sum of the percentages of separated water using the procedure in three steps was 66.0%, while the percentage of separated water when all additives were added together was 51.0% for the emulsion without the EOR polymer.

**Table 3:** Comparison between percentages of separated water by adding chemicals individually (3h test) and together (1 h and 3h tests) at low concentration, preparing emulsions without EOR polymer, with SPAM or HPAM

	Percentages of total separated water (%)		
	Chemicals added in 3 steps	Chemicals added together	
	(3 h test)	(1 h test)	(3 h test)
**Without EOR polymer**	66.0 ± 2.0	49.0 ± 3.0	51.0 ± 3.0
**With SPAM**	63.5 ± 0.5	55.0 ± 1.0	58.0 ± 2.0
**With HPAM**	38.8 ± 2.8	32.0 ± 2.0	37.0 ± 3.0

The influence of polymers was confirmed when Procedure (B) was used. Emulsions without the EOR polymer and with SPAM produced similar total percentages of separated water (66.0% and 63.5%, respectively), and the emulsion with HPAM was more stable, presenting separated water of 38.8%.

#### 3.3.2 Chemicals Added Together at High Concentrations

The application of Procedure (A), using a high concentration of chemicals, produced separated water percentages of the emulsions without and with SPAM of 77.0% and 68.0%, respectively. The emulsion containing HPAM had a lower percentage of separated water (59.0%). Similar behavior was observed for the low concentrations of chemicals. These results confirmed the greater stability of the emulsions containing HPAM in brine.

The employment of Procedure (B) promoted a significant reduction in the percentage of water separated with SPAM or HPAM in comparison with emulsions without the EOR polymer: 59.2%, 49.4%, and 75.0%, respectively. Unlike what was observed for low concentrations of chemicals, the sum of percentages separated in the three steps was not higher than the percentage of water separated when adding all chemicals at the same time. Another difference between low and high chemical concentrations was the efficiency of the chemicals: at low concentration, the greatest amount of water was separated after the second step, while the greatest amount of water was separated in the first step when using a high concentration of *D*_1_. Figure 4 shows the values of separated water in Procedure (A) and in each step of Procedure (B) involving the chemicals together at high concentrations without and with SPAM or HPAM.

**Figure 4:**
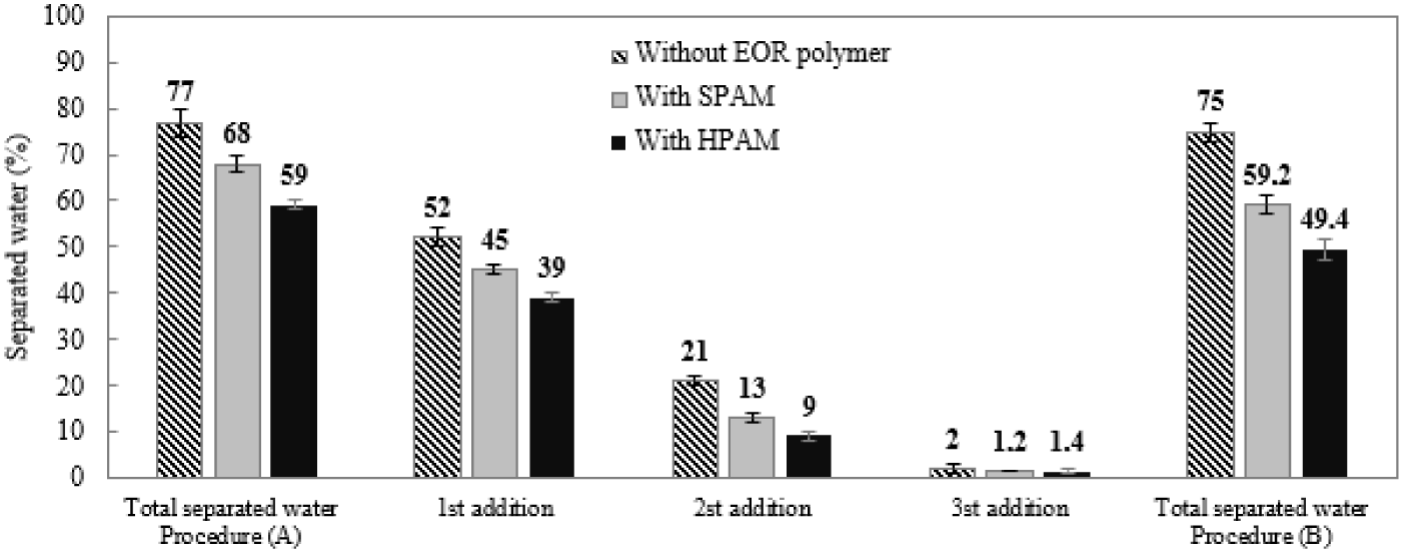
Percentage of water separated for emulsions submitted to Procedure (A) (chemicals added together) and Procedure (B) (chemicals added by steps), at high concentrations of chemicals, without and with 300 ppm of EOR polymers.

The analyses with high concentrations of chemicals produced water containing enough oil to be perceived with the naked eye. It is clear that the separated water from the emulsion containing SPAM, according to Procedure (B) (chemicals added by steps), had the worst quality, followed by the water separated from the emulsion containing SPAM using the same chemical addition procedure.

It is also important to note that in the chemical combination using Procedure (A), the water separation percentages without and with SPAM and HPAM were 77.0%, 68.0%, and 59.0%, respectively, which are very similar to those when *D*_3_ was used individually (Figure 1): 78.0%, 72.0%, and 59.0%, respectively. With Procedure (B), in the presence of SPAM and HPAM, the sums of percentages of separated water were 59.2% and 49.4%, respectively. Thus, the emulsions were more stable than those adding D_3_ individually (respectively 72.0% and 59.0%).

The TOG behavior was the same as that observed for chemicals added individually. TOG increased in the following order: samples without the EOR polymer < samples with HPAM < SPAM. Nevertheless, the TOG values were drastically higher when the chemicals were added in three steps for the emulsions containing polymer (SPAM or HPAM).

Between the emulsions without the EOR polymer in Procedures (A) and (B), the TOG values were 37.18 and 36.24 ppm, respectively. In the presence of SPAM, the values were 56.13 and 601.21 ppm, respectively. The TOG value was extremely high for Procedure (B), where the chemicals were added in three stages. In this test, manual agitation was performed after each addition of chemical for 1 min, and the water separated in each step was not removed from the conical tube due to the difficulty in removing a small volume of water from the tube. This manual agitation of the two phases in the system containing polymer, which interacted with the oil, may have facilitated the retention of more oil in the water compared with Procedure (A), where the chemicals were added all at once. With HPAM, the TOG values were lower than with SPAM in Procedure (A) and Procedure (B): 41.58 and 409.55 ppm, respectively. Figure 5 depicts the reduction in water quality also observed for the TOG values.

**Figure 5:**
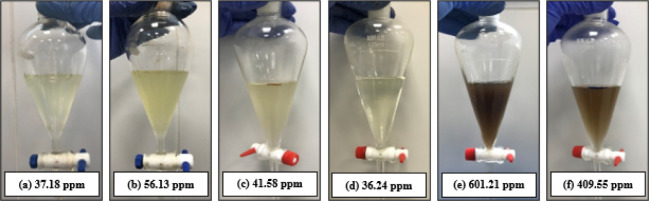
Water separated at the end of the bottle test with production chemicals added according to Procedure (A): (a) without EOR polymer, (b) with SPAM, and (c) with HPAM; and according to Procedure (B): (d) without EOR polymer, (e) with SPAM, and (f) with HPAM, with additional production chemicals.

Based on the data obtained, significantly high TOG values was observed, especially in the presence of EOR polymers, exceeding the daily limit of 29 ppm stipulated by Brazilian legislation, with peaks reaching up to 42 ppm.^57^ This scenario highlights the need for additional steps in water treatment in order to mitigate potential impacts associated with discharge into the marine environment or the reinjection of water in rescue processes. Furthermore, the increase in TOG levels implies an increase in operational costs since it requires the application of more efficient and expensive technologies to adapt the water quality to the limits required for discharge or reuse.

### 3.4 Interfacial Tension Analysis by Force Tensiometry

We compared the stability results of emulsions by the bottle test with interfacial tension analyses (Table 4).

**Table 4:** Droplet Size, Interfacial Tension, and Percentage of Separated Water for Emulsion 50/50 (W/O) without and with Chemicals, at Low and High Concentrations, in the Presence and Absence of SPAM or HPAM

				Particle size (μm)			
Chem	Conc (ppm)	EOR polymer	Separated water volume (%)	*D*(10)	*D*(50)	*D*(90)	Interfacial tension (mN/m)
-	-	-	0.0 ± 0.0	24.8 ± 2.8	109.5 ± 0.5	260.5 ± 7.5	27.64 ± 0.37
		SPAM	0.0 ± 0.0	26.4 ± 0.4	106.0 ± 6.0	292.5 ± 15.5	26.16 ± 0.42
		HPAM	0.0 ± 0.0	25.0 ± 1.0	81.4 ± 0.7	279.0 ± 3.0	22.04 ± 0.25
***D***_**1**_	50	-	8.5 ± 0.5	19.6 ± 12.9	115.5 ± 5.5	507.5 ± 31.5	18.32 ± 0.09
		SPAM	7.0 ± 0.0	31.2 ± 5.9	138.5 ± 15.5	505.0 ± 27.0	17.13 ± 0.46
		HPAM	3.0 ± 1.0	26.4 ± 0.6	90.7 ± 1.8	336.0 ± 8.0	19.96 ± 0.66
	650	-	55.0 ± 3.0	20.8 ± 14.9	120.2 ± 21.8	1,495.0 ± 45.0	13.24 ± 0.43
		SPAM	47.0 ± 3.0	44.7 ± 0.3	191.5 ± 7.5	1,245.0 ± 25.0	12.19 ± 1.43
		HPAM	39.0 ± 3.0	36.4 ± 3.2	145.5 ± 20.5	1,125.0 ± 5.0	11.35 ± 0.62
***D***_**2**_	65	-	7.5 ± 1.5	4.1 ± 0.5	81.0 ± 0.7	635.5 ± 3.5	17.77 ± 0.80
		SPAM	14.0 ± 2.0	41.2 ± 4.6	187.0 ± 25.0	765.5 ± 17.5	16.89 ± 0.54
		HPAM	4.5 ± 1.5	26.7 ± 0.7	94.6 ± 4.4	382.0 ± 12.0	17.97 ± 0.70
	845	-	58.0 ± 2.0	10.0 ± 4.8	85.7 ± 6.1	1,590.0 ± 40.0	13.00 ± 0.70
		SPAM	52.0 ± 1.0	29.6 ± 0.5	183.0 ± 18.0	1,295.0 ± 25.0	12.70 ± 0.42
		HPAM	45.0 ± 1.0	39.1 ± 2.5	163.0 ± 5.0	1,175.0 ± 55.0	11.31 ± 0.01
***D***_**3**_	50	-	24.0 ± 0.0	55.5 ± 7.7	210.5 ± 28.5	956.5 ± 53.5	14.04 ± 0.23
		SPAM	24.0 ± 0.0	42.7 ± 2.8	191.5 ± 3.5	934.5 ± 38.5	12.50 ± 0.44
		HPAM	14.0 ± 2.0	40.3 ± 8.4	139.0 ± 28.0	776.0 ± 13.0	13.58 ± 0.69
	650	-	78.0 ± 2.0	5.8 ± 0.4	578.0 ± 117.5	1,855.0 ± 45.0	11.61 ± 0.13
		SPAM	72.0 ± 2.0	25.3 ± 0.3	180.5 ± 13.5	1,690.0 ± 70.0	9.99 ± 0.54
		HPAM	59.0 ± 1.0	51.6 ± 1.2	206.0 ± 3.0	1,435.0 ± 5.0	9.46 ± 0.31
***I***_**cor**_	80	-	0.0 ± 0.0	18.4 ± 0.3	55.9 ± 0.1	208.5 ± 0.5	23.66 ± 0.28
		SPAM	0.0 ± 0.0	29.5 ± 0.4	96.0 ± 1.4	294.0 ± 5.0	24.30 ± 0.80
		HPAM	0.0 ± 0.0	35.7 ± 3.2	111.0 ± 9.0	286.5 ± 2.5	22.14 ± 0.48
	1040	-	0.0 ± 0.0	32.9 ± 1.0	116.0 ± 3.0	264.0 ± 7.0	22.77 ± 0.59
		SPAM	0.0 ± 0.0	40.4 ± 4.0	124.0 ± 10.0	300.0 ± 5.0	20.85 ± 0.53
		HPAM	0.0 ± 0.0	33.3 ± 0.8	122.5 ± 5.5	335.5 ± 10.5	21.59 ± 0.43
***I***_**sca**_	40	-	0.0 ± 0.0	23.8 ± 0.3	75.9 ± 0.2	189.5 ± 2.5	22.69 ± 0.45
		SPAM	0.0 ± 0.0	30.1 ± 0.3	99.7 ± 1.3	300.0 ± 2.0	21.39 ± 0.69
		HPAM	0.0 ± 0.0	37.4 ± 1.9	116.0 ± 4.0	286.0 ± 1.0	20.69 ± 0.69
	520	-	0.0 ± 0.0	38.3 ± 1.7	105.5 ± 2.5	241.0 ± 2.0	21.40 ± 0.31
		SPAM	0.0 ± 0.0	39.8 ± 1.0	125.5 ± 0.5	305.5 ± 9.5	19.24 ± 0.52
		HPAM	0.0 ± 0.0	33.4 ± 0.1	120.5 ± 0.5	352.0 ± 15.0	22.43 ± 0.37
**All additives together**	285	-	49.0 ± 3.0	24.5 ± 2.6	942.0 ± 46.0	1,470.0 ± 80.0	13.10 ± 0.12
		SPAM	55.0 ± 1.0	58.9 ± 0.2	286.0 ± 13.0	1,320.0 ± 100.0	12.19 ± 1.59
		HPAM	32.0 ± 2.0	39.9 ± 11.5	153.0 ± 53.0	1,145.0 ± 45.0	11.78 ± 0.04
	3705	-	77.0 ± 3.0	36.6 ± 4.8	1,024.5 ± 25.5	1,935.0 ± 25.0	10.44 ± 0.28
		SPAM	68.0 ± 2.0	24.8 ± 1.6	209.0 ± 3.0	1,455.0 ± 75.0	8.38 ± 0.01
		HPAM	59.0 ± 1.0	36.5 ± 4.1	220.0 ± 31.0	1,505.0 ± 65.0	8.16± 0.04

#### 3.4.1 Aqueous Phase without EOR Polymer

The interfacial tension results corroborated the bottle test results: *D*_1_ and *D*_2_ presented similar performance (∼8.0%) and provoked similar interfacial tension (∼18.0 mN/m), while *D*_3_ presented the best performance (24%) and promoted lower interfacial tension (∼14 mN/m) than *D*_1_ and *D*_2_. This tendency was observed for both low and high concentrations of chemicals. The literature mentions that when comparing the mechanism of action of demulsifiers, a decrease in interfacial tension is observed. In this context, the additives migrated to the water–oil interface, thus displacing the stabilizing substances and, therefore, facilitating the breakdown of the emulsion through the least repulsive and/or sterically impeded type of interface.^58–61^ Despite this, the interpretation of the results requires a joint analysis of the data, since the reduction in measured interfacial tension may be associated with the presence of interfacially active species that affect intrinsic parameters of the technique, such as the viscosity of the system, without necessarily reflecting the destabilization of the emulsion. In the presence of EOR polymers, for example, the increase in the viscosity of the aqueous phase may interfere with the oscillation dynamics of the measuring accessory, introducing variations in the experimental signal and influencing the apparent values of interfacial tension. Thus, the relationship between demulsification efficiency and interfacial tension should be established for the system under the same conditions, for example, with or without EOR polymer.

When the chemicals were added at high concentrations without the EOR polymer, interfacial tension values very similar to those of the low concentration of chemicals were observed for *I*_sca_ and *I*_cor_, confirming the weak activity of these chemicals at the interface. On the other hand, very significant reductions were observed (from 27.64 mN/m without chemicals to 11.61, 13.00, and 13.24 mN/m when using *D*_3_, *D*_2_, and *D*_1_, respectively). These values also agreed with the percentages of separated water: 78.0, 58.0, and 55.0 for *D*_3_, *D*_2_, and *D*_1_, respectively. The similarity in the results for *D*_2_ and *D*_1_, observed at low concentrations, was confirmed at high concentrations. A lower interfacial tension value was observed for the addition of all chemicals together at high concentration (10.44 mN/m), with a percentage of water separated of 77.0%. This also confirmed the relationship between interfacial tension and the percentage of separated water because *D*_3_ presented similar values for both parameters. At this concentration, a synergistic effect of the corrosion inhibitor, scale inhibitor, and demulsifiers was not observed, since the percentage of water separated for all chemicals together (77.0%) was very similar to that obtained when adding *D*_3_ individually (78.0%).

#### 3.4.2 Aqueous Phase Containing EOR Polymers

The interfacial tension of the samples without the EOR polymer but with SPAM and HPAM was 27.64, 26.16, and 22.04 mN/m, respectively. This result showed that HPAM has a higher tendency to move to the interface than SPAM. This behavior may be associated with specific chemical interactions between the functional groups of HPAM (amide and hydroxyl groups) and asphaltenes, including hydrogen bonds with asphaltene heteroatoms, as well as electrostatic interactions with charged asphaltic sites. Studies conducted by Liu et al. (2021) and Liu et al. (2022), for example, demonstrated the effective interaction between HPAM and asphaltenes, resulting in the formation of a carboxy-asphaltene-amide composite unit.^62,63^ Furthermore, this unit showed a high potential for the formation of a flexible interfacial film in water/oil (w/o) emulsions. Based on these results, it is believed that, in the presence of asphaltene-rich systems, such as heavy oil samples, the formation of the polymer-asphaltene complex is more favored, leading to the development of an interfacial layer. Consequently, steric hindrance and increased interfacial stiffness restrict the access of emulsion-destabilizing molecules to the interface, resulting in a significant reduction in the destabilization efficiency. On the other hand, although SPAM presents similar characteristics to HPAM, its more voluminous side chain may reduce the number of available interaction sites, hindering a more effective interaction with asphaltenes. As a result, an interfacial layer with a less structured and cohesive organization is formed, reducing the impact on the demulsification process compared to that observed for HPAM.

For chemicals at low concentration with SPAM, a slight reduction in interfacial tension was observed when *I*_sca_ and *I*_cor_ were added: from 26.16 (sample without chemicals) to 21.39 and 24.30 mN/m, respectively. The reduction in interfacial tension when *I*_sca_ and *I*_cor_ were added was also observed for the sample without the EOR polymer, which showed a slight tendency of these chemicals to move to the interface, reducing interfacial tension but not acting as emulsion breakers. As expected, a greater reduction was observed when demulsifiers were added. *D*_3_ promoted the greatest reduction (from 26.16 to 12.50 mN/m). The interfacial tension values for *D*_2_ and *D*_1_ were similar (16.89 and 17.13 mN/m, respectively). These results agree with the percentage of separated water (24.0%, 14.0%, and 7.0% for *D*_3_, *D*_2_ and *D*_1_, respectively). In general, the results obtained with SPAM were very similar to those of the samples without the EOR polymer when using the low concentration of chemicals.

When used demulsifier at high concentrations was used, the percentages of phase separation were slightly lower with SPAM (72.0%, 52.0%, and 47.0%) than without the EOR polymer (78.0%, 58.0%, and 55.0%) for *D*_3_, *D*_2_ and *D*_1_, respectively. However, the interfacial tension reductions were very similar for both samples. In this case, we suggest that the difference observed in the demulsifier performance was related to a kinetic effect during the demulsification process. In the presence of SPAM at high concentration, no synergistic effect was observed between the chemicals, as was also observed for the sample without the EOR polymer.

For chemicals at low concentration with HPAM, no reduction in interfacial tension was observed when *I*_sca_ and *I*_cor_ were added: from 22.04 (sample without chemicals) to 20.69 and 22.14 mN/m, respectively; unlike what was observed for the samples without and with SPAM, where there was a slight reduction in tension. The presence of HPAM at the interface makes it difficult for these chemicals to migrate to the interface.

Also, as expected, greater interfacial tension reduction was observed when the demulsifiers were added. *D*_3_ promoted the largest reduction (from 22.04 to 13.58 mN/m). The interfacial tension values for *D*_2_ and *D*_1_ were similar (17.97 and 19.96 mN/m, respectively). These results were correlated with the percentage of separated water (14.0%, 4.5%, and 3.0% for *D*_3_, *D*_2_, and *D*_1_ respectively). In general, the reduction of interfacial tension and the percentage of separated water with HPAM were smaller in comparison with the samples without polymer and with HPAM when using low concentration of chemicals.

When the chemicals were added at high concentration, interfacial tension values very similar to those at low concentration were observed for *I*_sca_ and *I*_cor_. In this case, *I*_sca_ and *I*_cor_ did not show any action at the interface. On the other hand, very large reductions were observed, from 22.04 mN/m without chemicals to 9.46, 11.31, and 11.35 mN/m when using *D*_3_, *D*_2_ and *D*_1_, respectively. These values were also correlated with the percentage of separated water (59.0%, 45.0%, and 39.0% for *D*_3_, *D*_2_ and *D*_1_, respectively). The similarity of the results for *D*_2_ and *D*_1_, observed at low concentration, was confirmed at high concentration. When the chemicals were added together, the percentage of separated water and interfacial tension were very similar to those of *D*_3_ individually, with no synergistic effect being observed again at the highest concentration of chemicals.

### 3.5 Size and Size Distribution of Droplets by Laser Diffraction

Based on knowledge gained from physicochemical studies of emulsion interfaces, it is known that the size of the droplets is inversely proportional to the stability of the system. The increase in droplet size can cause collisions between the droplets of the dispersed phase due to the Brownian motion of the molecules, which consequently causes coalescence followed by phase separation.^64,65^

We performed droplet size measurements of the emulsions just after they were prepared, after resting for 20 min in the thermostatic bath at the test temperature, and again at the end of the bottle test, which was carried out at 100 °C. The results are reported in Table 4. With regard to *D*(90), the w/o emulsion without chemicals presented almost the same droplet size distribution before and after resting in the thermostatic bath, and an increase in droplet size after the bottle test (from ∼160 to ∼260 μm). With SPAM, an increase in droplet size from before resting to after the bottle test (from ∼166 to ∼292 μm) was observed. These values were slightly higher than those for the sample without the EOR polymer. Similar behavior was observed with HPAM, showing an increase in droplet size from before resting to after the bottle test, in this case, from ∼171 to ∼279 μm. Based on these results, it was decided to evaluate the emulsions containing chemicals at low and high concentrations only at the end of the bottle test (60 min), because the values of droplet size just after preparing the emulsion and after resting in the bath were very similar, and the chemical was added after the emulsion’s resting.

Without the EOR polymer at low concentrations, *I*_sca_ and *I*_cor_, which did not provoke phase separation, did not induce an increase in droplet size, as expected. *D*_1_ and *D*_2_, which presented a very similar percentage of separated water (∼8.0%), also presented a very similar droplet size, especially in relation to *D*(90), which was 507.50 and 635.5 μm, respectively. *D*_3_, with the highest efficiency in separating water (24.0%), induced the largest droplet size (*D*(90), 956.50 μm), evidencing its efficiency in promoting the droplets’ coalescence. As expected, all chemicals together, with water separation of 49.0%, presented the highest droplet sizes (*D*(50) 942.00 μm and D(90) 1,470.00 μm), corroborating the good relationship between the percentage of separated water and droplet size.

Without the EOR polymer at high concentrations, larger droplet sizes were observed, associated with a higher percentage of separated water compared to the results for lower dosages of chemicals. As observed for low concentrations of the chemicals, *D*_1_ and *D*_2_ presented droplet sizes in the same range, in agreement with the similar percentages of separated water. *D*_3_, which promoted the highest percentage of separated water (78.0%), also presented both *D*(50) 578.00 μm and *D*(90) 1,855.00 μm, relatively larger than the emulsion containing other chemicals. The addition of all chemicals together at the highest concentration (3705 ppm) resulted in significantly higher values of *D*(50) (1,024.50 μm) and *D*(90) (1,935.00 μm), which was expected since the addition of chemicals at high concentration had increased the coalescence of the droplets.

With SPAM at low concentration, *I*_sca_ and *I*_cor_ did not promote phase separation and also did not induce an increase in droplet sizes, as expected, in comparison with the emulsion without chemicals. *D*_1_ and *D*_2_, which promoted similar percentages of separated water without the EOR polymer (∼8.0%), presented different values when SPAM was added (7.0% and 14.0%). This was correlated with the *D*(90) values. For *D*_2_, the droplet size was higher (765.50 μm) than that for *D*_1_ (505.00 μm). In turn, *D*_3_, with the highest efficiency in separating water (24.0%), exhibited the largest droplet size (*D*(90) 934.50 μm), evidencing the efficiency in promoting the droplets’ coalescence, presenting the same value of separated water and droplet size as the sample without the EOR polymer. As expected, all chemicals together, with separated water of 55.0%, promoted the largest droplet size (D(90) 1,320.00 μm).

With SPAM at high concentrations of chemicals, larger droplet sizes were observed, associated with a higher percentage of separated water than the results for lower dosages of chemicals. Without the EOR polymer, *D*_1_ and *D*_2_ presented similar water separation values (47.0% and 52.0%) and droplet sizes (1,245.00 and 1,295.00 μm), respectively. *D*_3_, which presented the highest percentage of separated water (72.0%,) also presented *D*(90) of 1,690.00 μm, relatively larger than the emulsion containing other chemicals. The addition of all chemicals together at the highest concentration (3705 ppm) promoted similar values of *D*(50) 209.00 μm and *D*(90) 1,455.00 μm when compared to the *D*_3_ sample individually (*D*(50) 180.50 μm and *D*(90) 1,690.00 μm). This was positively correlated with the percentage of separated water and was also similar (68.0% and 72.0%) for the chemicals together and *D*_3_ individually, respectively.

When applied individually, the demulsifiers alone promoted significant phase separation. Scale and corrosion inhibitors, as well as the EOR polymers, had no measurable influence on the volume of water separated. In stepwise addition, the concentration of chemicals proved to be a determining factor. At low concentrations, the order of addition affected separation, particularly in the presence of HPAM and SPAM. At high concentrations, the order of addition had no significant influence, and similar separation volumes were obtained regardless of the presence of the EOR polymers. In three-stage tests, highly concentrated systems promoted phase separation after the first stage, while low-concentration systems required at least two stages for effective demulsification. Additional tests confirmed that corrosion and scale inhibitors had no synergistic effect with other additives and did not contribute to water separation. Water quality assessments revealed that the EOR polymers increased TOG levels, with results following the same order: without EOR polymer < HPAM < SPAM. This effect was more intense when the additives were introduced in three steps. These findings indicate that the type, concentration, and addition sequence of production chemicals significantly influence emulsion stability and the quality of the separated water.

Based on this, you can relate both results, including the interfacial tension. Figure 6 shows a set of results related to demulsifier *D*_1_ at high concentration (650 ppm), with and without SPAM and HPAM. The reduction in the volume of separated water is accompanied by a decrease in droplet size and interfacial tension. This behavior was observed for all demulsifiers (*D*_1_, *D*_2_ and *D*_3_). Analysis shows that the addition of EOR polymers promotes greater occupation of the water–oil interface due to their amphiphilic nature, intensifying interfacial competition. Consequently, the demulsifier’s ability to displace interfacially active species is reduced, limiting the coalescence of water droplets. This effect results in smaller average droplet diameters and, consequently, a smaller volume of separated water at the end of the test, characterizing a decrease in the demulsifier’s efficiency.

**Figure 6:**
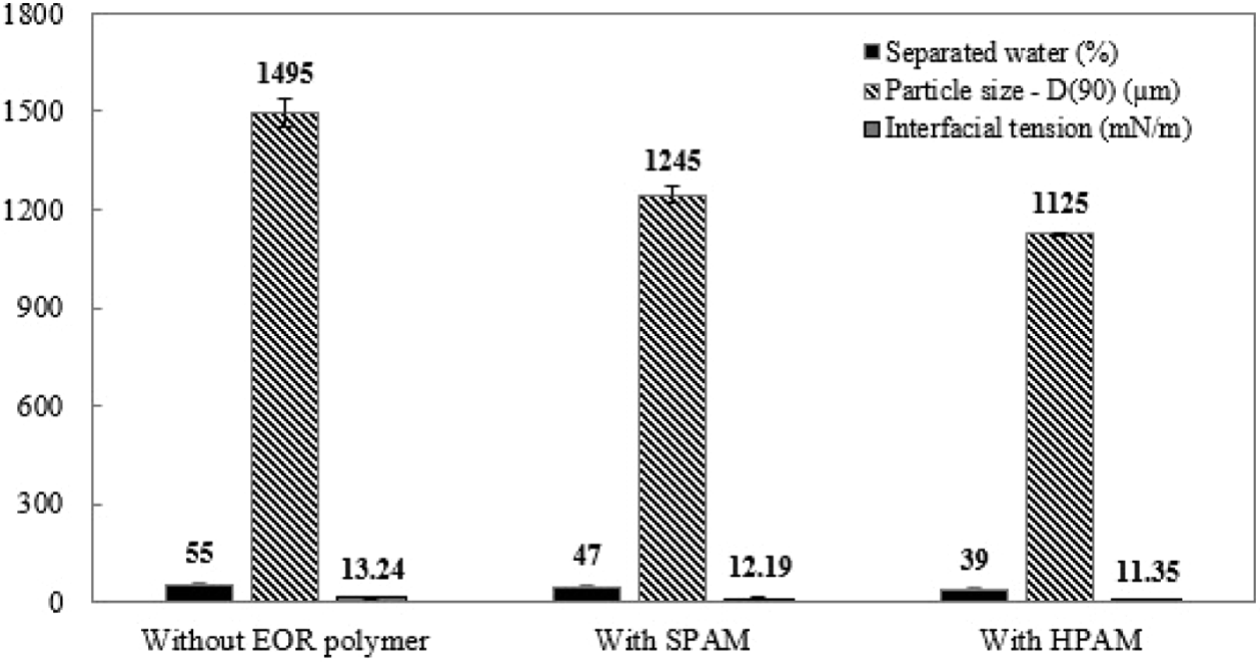
Correlation among percentage of separated water (black), particle size (black hatched gray bars), and interfacial tension (gray). The tests involved demulsifier D_1_ at high concentration (650 ppm), with and without SPAM or HPAM.

With HPAM at a low concentration, *I*_sca_ and *I*_cor_, which did not promote phase separation, also did not induce an increase in droplet sizes, as expected, in comparison with the emulsion without chemicals. *D*_1_ and *D*_2_, which presented very similar water separation percentages and droplet sizes without the EOR polymer, also presented similar values in the presence of HPAM. In the presence of HPAM, the droplet size values were much smaller, as well as the percentage of separated water. This corroborated the greater stability of the emulsions in the presence of HPAM. The effect has also been associated with the interfacial tension, which was smaller. *D*_3_, with the highest efficiency in separating water (14.0%), induced the larger droplet size (*D*(90) 776.00 μm), evidencing the efficiency in promoting the droplets’ coalescence. As expected, all chemicals together, with a water separation percentage of 32.0%, presented the largest droplet sizes (D(90) 1,145.00 ± 45.00 μm).

With HPAM at high concentrations, larger droplet sizes were observed, associated with a higher percentage of separated water compared to the results for lower dosages of chemicals. For the highest concentration of chemicals, as observed for samples without and with SPAM, *D*_1_ and *D*_2_ achieved similar percentages of separated water (39.0% and 45.0%) and droplet size (1,125.00 μm and 1,175.00 μm), respectively. *D*_3_, which presented the highest percentage of separated water (59.0%), also presented *D*(90) 1,435.00 μm, relatively larger than the emulsion containing other chemicals. The addition of all chemicals together at the highest concentration (3705 ppm) presented similar values of *D*(50) 220.00 μm and *D*(90) 1,505.00 μm when compared to the D_3_ individually added (*D*(50) 206.00 μm and *D*(90) 1,435.00 μm). The percentage of separated water was the same (59.0%).

The close relationship between the percentages of separated water and the size of the droplets evidenced the consistency of the results.

## 4 Conclusions

The influence of production chemicals, individually and together, on the performance of the demulsifier and the quality of the separated water was evaluated using a synthetic emulsion composed of brine and heavy oil. The EOR polymers affected the performance of the demulsifiers. HPAM had the greatest influence, possibly due to its higher molar mass and greater expansion in comparison with SPAM. Scale and corrosion inhibitors, when added individually, did not increase the efficiency of phase separation, while their addition in combination with the other chemicals did not influence the performance of the demulsifier. Between the two methods of adding the chemicals (together or in three stages), the stability of the emulsion decreased when the method used was in three stages. At low concentrations of the chemicals, this behavior was not observed, but it was observed for the high concentration of the chemicals. At low concentration, the highest water separation occurred during the the second step, whereas at higher concentrations most of the water was already separated during the first step. Additionally, regarding the quality of the separated water, the EOR polymers had a negative impact. The quality of separated water decreased in the following order: without the EOR polymer > with HPAM > with SPAM. This effect was more pronounced when the additives were introduced in three steps, in which the system was stirred in the presence of the EOR polymers. The negative impact of EOR polymers on water quality highlights the need for operational adjustments for the combined use of additives to avoid impacts on the discharge or reuse of produced water. The good relationship between the percentages of separated water, interfacial tension, and droplet size demonstrated the consistency of the results.
